# Antiseptic Treatment of Typhoid Fever

**Published:** 1891-09-26

**Authors:** 


### THE PRACTITIONER'S MIRROR AND RETROSPECT.

[*The Editor will be glad to receive offers of co-operation and contributions from members of the profession. All letters should be addressed to* THE EDITOR, THE LODGE, PORCHESTER SQUARE, LONDON, W.]

### MEDICINE.

### ANTISEPTIC TREAMENT OF TYPHOID FEVER.

It is difficult to determine how much of the newly introduced methods of treating typhoid fever may be described as new treatment, and how much as old treatment reintroduced. We have recently drawn attention to the results of treatment of enteric fever by cold baths. But the recent advances in bacteriology have tended to prove that the materies morbi of typhoid fever is a microbe, and the situation of the lesions being comparatively accessible, it would appear to lend itself to treatment by germicidal remedies.

The germicidal treatment of course assumes that the disease is caused by a germ, but although most bacteriologists and many continental authorities are satisfied on this point, English pathologists still regard it as an open question. The identification of the typhoid germ is, at any rate, unsettled, but the same difficulties have been encountered with regard to the cholera microbe, and though "the bacillus of Eberth is regarded as the specific cause of enteric fever, it is difficult to distinguish it from a number of minute organisms which are found in water. As many as ten bacilli have been noted, which are similar, and of these at least three are scarcely distinguishable from the fever - bearing germ. Their recognition depends upon minute differences which are not easily conveyed by description. Bacteriologists have tried to erect criteria between the real and the pseudo-typhoids, the typhus-änliche bacillen, (1) by the manner of moving of the typhoid bacillus; (2) by the difficulty of staining it, and by its discolouration after a method devised by Gram; and (3) by the way the bacillus propagates on the potatoe."* Some interesting observations and experiments by Holz are published in his Zeitschrift für Hygiene, viij. 1890.

In a recent lecture Dujardin-Beumetz† states that the putridity of the intestines plays a considerable rôle in typhoid fever. This putridity results from the particular state of the digestive tube, and the numerous ulcerations which develop there, resulting sometimes even in a sphacelus of a portion of the mucosa. We are especially indebted to Bouchard for enlightenment as to the best means of inducing intestinal antisepsis in typhoid fever. Bouchard first proposed charcoal, then iodoform, then naphthalin, finally napthol, and it must be admitted, says the Professor, that the latter agent is much superior to those first mentioned. There are two varieties of naphthol — $\alpha$-naphthol and $\beta$-naphthol. The former of these is stated to be more soluble, less toxic, but more irritant; the latter less soluble, more toxic, but less irritant.

Bouchard has given preference to $\beta$ naphthol, which he administers in association with salicylate of bismuth. In reference to the use of $\beta$ naphthol in typhoid fever, Professor Burney Yeo reminds us that it has many supporters, such as Petresco, of Bucharest, who has borne valuable testimony to the efficacy of this drug. He had experimented with carbolic acid, salicylic acid, benzoic acid, kairin, calomel, corrosive sublimate, and boric acid, and without any favourable results. He then tried a saturated solution of sulphide of carbon, with which he was much better pleased; and lastly he tried naphthol, fifteen grains three times a day, and he reports that he had results more favourable than with any other remedy, that the rate of mortality was reduced, and the course of the disease favourably modified. Dr. Mitchell Clarke, of Bristol, has also recorded some observations on the use of $\beta$ naphthol and also of hydronaphthol in typhoid fever, and he thus summarises his results from the use of naphthol in typhoid fever: 1. A reduction in the average duration of the fever. 2. Stools became much less offensive. 3. A diminution of abdominal tenderness and meteorism. 4. Early cleansing of tongue and less dryness of mouth and lips. 5. Absence of albuminuria. 6. Convalescence more rapid and strength less reduced. 7. Less risk of propagating the disease to others. 8. Tendency to secondary complications diminished. Dr. Burney Yeo, speaking of his own results says: "I could bring before you the histories of many other cases, but they all tell the same story—great modification of the temperature curve, deodorisation of the motions, cleansing of the tongue, maintenance of a comparative well-being, and much less evidence of a septic state of blood, rapid convalescence, and much less loss of strength than is ordinarily observed after an attack of typhoid fever. . . . In commending this method of treating typhoid fever to your notice as supporting the idea of the value both of general and local antisepsis in this disease, I am far from wishing you to conclude that this is the very best method to follow in all cases, or that a more perfect antiseptic treatment may not be attainable. It seems probable from recent observations that the various antiseptic methods may be adopted with advantage, and that some constitutions may be influenced more readily and favourably by one method than by another."

* *London Medical Record*, February 20th, 1891.
† *Therapeutical Gazette*, January, 1891.

Dr. F. Schmidt*, in his graduation thesis at Berne, in 1889, reports the results which he obtained in his employment of thallin in twenty-two cases of typhoid fever, the remedy being given in doses varying from three-quarters to three grains in a day, with nothing given at night, and the following are his conclusions: 1. The mortality of typhoid fever treated by thallin is less than that obtainable by any other mode of treatment. Thallin, in the doses above-mentioned, distinctly reduces the temperature in cases of moderate intensity, but in typhoid fever of extreme gravity the dose is insufficient. It also would seem that the patients support thallin better than cold baths. 3. In general the duration of this disease is not diminished, although this effect would appear to occur in a few isolated cases. 4. No unfavourable secondary action was noted on the heart or lungs. There was no collapse or irritation of the kidneys. Nevertheless, basing his conclusions on the results obtained by other authors, Schmidt advises the withholding of thallin in all cases where renal lesions have been detected. 5. Thallin maintains a favourable influence on the sensorium in all cases of typhoid fever except those of extreme gravity. 6. Complications and relapses are not prevented by the use of thallin any more than by any other form of treatment. 7. If it is impossible to discover any specific action of thallin on typhoid fever, at least it would appear that certain effects exist which render this action probable.

Of calomel in typhoid fever we have the recent experience of Dr. W. H. Broadbent, who relates the following case in which the recognition of ptomain poisoning from the intestinal canal explained obscure and dangerous symptoms, and was the clue to effectual treatment. The case was one of enteric fever seen at about the fourteenth day of the disease. The initial symptoms had been fairly characteristic, the temperature ranged low, there was no distension of the abdomen, the bowels were constipated, and there was extreme prostration and constant vomiting of greenish liquid. The patient was so weak that he could not turn in

*"Les Nouveux Remèdes," July 24, 1890. *Ther. Gaz.*, September 15, 1890.

bed, and his nervous prostration was extreme. The pulse was very frequent, extremely small, short, and weak; the feet and knees were cold and clammy; and his hands were swollen, cold, and livid. It was obvious that the nervous system was overwhelmed by some poison, and probably such poison was manufactured in the intestine by bacteria and absorbed. Clearly, the patient's one chance was that the bacteria and their products should be swept from the alimentary canal. The intestinal lesions fortunately could, in this case, be disregarded, there being no local tenderness, no abdominal distention, no intestinal catarrh. Three grains of calomel were therefore administered, as best suited to serve the double purpose just mentioned. The next day the patient was relieved, all the unfavourable symptoms cleared away, and the case pursued a normal course, and ended in recovery.

It is very probable, indeed, that the beneficial results from the adoption of some intestinal antiseptic method in typhoid fever are due to the destruction of the putrifactive bacteria, and thus to preventing the absorption of ptomaines, rather than to the actual destruction of the specific germs of enteric fever. At any rate, there seems to be such an almost universal consensus of opinion as to the wholly beneficial results of such methods, that it is difficult to conceive that the reports are not based on sound conclusions. As to the relative value of the different antiseptics, the investigations in each case are too meagre as yet to determine to which of the many advocated we should pin our faith.

